# Post-natal erythromycin exposure and risk of infantile hypertrophic pyloric stenosis: a systematic review and meta-analysis

**DOI:** 10.1007/s00383-016-3971-5

**Published:** 2016-09-21

**Authors:** L. Murchison, P. De Coppi, S. Eaton

**Affiliations:** 1 Stem Cells and Regenerative Medicine Section, Developmental Biology and Cancer Programme, UCL Great Ormond Street Institute of Child Health, 30 Guilford Street, London, WC1N 1EH UK; 2 Great Ormond Street Hospital for Children, London, UK

**Keywords:** Pyloric stenosis, Erythromycin, Macrolide antibiotics

## Abstract

**Purpose:**

Macrolide antibiotics, erythromycin, in particular, have been linked to the development of infantile hypertrophic pyloric stenosis (IHPS). Our aim was to conduct a systematic review of the evidence of whether post-natal erythromycin exposure is associated with subsequent development of IHPS.

**Methods:**

A systematic review of postnatal erythromycin administration and IHPS was performed. Papers were included if data were available on development (yes/no) of IHPS in infants exposed/unexposed to erythromycin. Data were meta-analysed using Review Manager 5.3. A random effects model was decided on a priori due to heterogeneity of study design; data are odds ratio (OR) with 95 % CI.

**Results:**

Nine papers reported data suitable for analysis; two randomised controlled trials and seven retrospective studies. Overall, erythromycin exposure was significantly associated with development of IHPS [OR 2.45 (1.12–5.35), *p* = 0.02]. However, significant heterogeneity existed between the studies (*I* ^2^ = 84 %, *p* < 0.0001). Data on erythromycin exposure in the first 14 days of life was extracted from 4/9 studies and identified a strong association between erythromycin exposure and subsequent development IHPS [OR 12.89 (7.67–2167), *p* < 0.00001].

**Conclusion:**

This study demonstrates a significant association between post-natal erythromycin exposure and development of IHPS, which seems stronger when exposure occurs in the first 2 weeks of life.

## Introduction

Infantile hypertrophic pyloric stenosis (IHPS) affects 1.9 of every 1000 live births [1] making the condition the most common cause of surgical intervention in the first 6 months of life [2]. IHPS is characterised by hypertrophy of the pylorus resulting in gastric outlet obstruction, leading to the infant presenting with projectile vomiting and severe dehydration.

Although genetics [3] and male sex [4] have been identified as risk factors, the aetiology of IHPS is largely unknown. Furthermore, changes in the incidence rates of IHPS have led to the hypothesis that environmental factors may have a role in the development of the condition [5].

Several studies have identified a strong relationship between exposure to erythromycin and development of IHPS [6]—with some studies identifying an eight to tenfold increase in risk of developing IHPS when erythromycin was administered in the first 2 weeks of life [7]. One theory is that erythromycin interacts with the receptors of motilin, an intestinal peptide that stimulates contraction of gut smooth muscle. This interaction could therefore produce contraction of the gastric and pyloric bulb, resulting in hypertrophy of the pylorus [8]. However, other studies refute the association between erythromycin treatment in infants and the development of IHPS entirely, identifying no association [9].

The aim of this study was to perform a systematic review and meta-analysis of published studies to clarify and quantify the relationship between any post-natal exposure to erythromycin and the development of pyloric stenosis. A second aim was to determine whether treatment with erythromycin within the first 2 weeks of life increased the magnitude of this association.

## Methods

A systematic literature search was performed of all studies published from 1 January 1970 and 1 July 2016, using PubMed, Ovid Medline, Embase and the Cochrane Library with the medical subject heading (MeSH) terms and text words: (infantile hypertrophic pyloric stenosis OR pyloric stenosis) AND (macrolide OR erythromycin) and similar variants. Search criteria were limited to studies published in the English language, and by age of subject (age less than 6 months) to ensure that only infantile cases of pyloric stenosis were included for analysis. Reference lists of included articles and abstract lists of relevant national and international meetings were also searched to identify other studies which could be included for analysis.

**Fig. 1:**
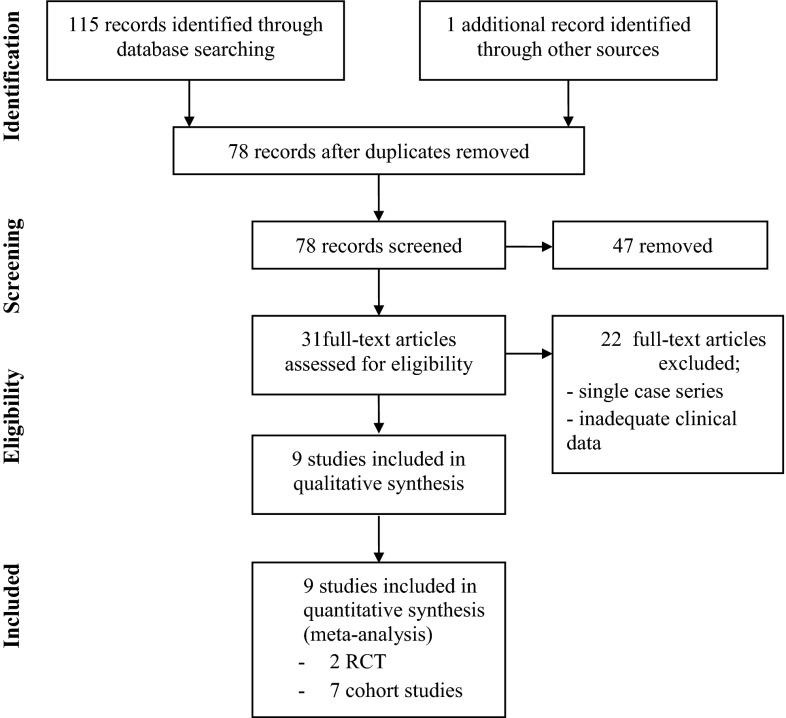
Diagram of workflow in the systematic review and meta-analysis

Studies were then assessed for inclusion by two authors independently (LM, SE). Our aim was to ensure that all robust studies which reported an association between erythromycin exposure and subsequent development of IHPS were included for analysis. Studies were excluded for several reasons; insufficient data available for analysis, unable to extract suitable data to allow meta-analysis, type of macrolide not explicitly stated, route of administration was only to the mother (either ante-natal or post-natal transfer in breast milk) or if route of administration of erythromycin was ambiguous. When more than one publication from an overlapping cohort was identified, the largest study with the most rigorous methodology was selected. Duplicate data, already available as a published paper, which had been published in the form of letters to the editor of journals was also excluded. The selection process is illustrated in Fig. 1. Data was independently extracted by the authors.

The meta-analysis was performed using Mantel–Haenszel random effects model using the Cochrane Collaboration’s Review Manager (RevMan 5.3, the Nordic Cochrane Centre, the Cochrane Collaboration, Copenhagen) to calculate the overall odds ratio (OR), 95 % confidence interval (CI) and *I* ^2^ test statistic for heterogeneity of studies.

Publication bias was assessed using the funnel plot method.

## Results

Literature search identified 115 papers for potential inclusion; 104 did not meet the criteria for inclusion and were excluded from the meta-analysis (Fig. 1). The remaining nine studies comprised two randomised control trials (one study on erythromycin used for improving enteral feeding tolerance and a second study on oral erythromycin for treatment of gastrointestinal dysmotility in preterm infants), and seven retrospective cohort studies. The characteristics of eligible studies are shown in Table 1.

**Table 1:** A summary of the studies included detailing country of origin, study type, data source, total number of infants studied, number of infants within study group who were exposed to erythromycin, and subsequently developed IHPS and the weight of the study in the meta-analysis

Study (year)	Country	Study type	Source of data	Total number of infants	Number of infants exposed to erythromycin who developed IHPS	Study weight (%)
Ng et al. (2001) [10]	China	Randomised control trial	Preterm infants admitted to the neonatal unit at Prince of Wales Hospital, Hong Kong from November 1998–May 2000	29	0	/
Cooper et al. (2002) [11]	United States	Cohort study	Medicaid or TennCare (Tennessee’s program for Medicaid enrollees and uninsured individuals) births in Tennessee from 1985–1997	306891	9	18
Eberly et al. (2015) [12]	United States	Cohort study	Infants born between 1 June 2001 and 1 April 2012 registered with the TRICARE Management Activity military health system (MHS) database	1069900	17	19.2
Mahon et al. (2001) [13]	United States	Cohort study	Infants born from 1 June 1993–31 December 1999, Wishard Hospital, Indianapolis	14407	4	15
Honein et al. (1999) [14]	United States	Cohort study	Infants born in a community hospital from January–February 1999	125	7	5.3
Ludvigsson et al. (2016) [9]	Sweden	Cohort study	Nationwide cohort of infants born between July 2005 and December 2010	582256	0	5.5
Ericson et al. (2015) [15]	United States	Cohort study	Infants from 348 NICUs managed by the Pediatrix Medical Group between 1997–2012	19001	10	17.9
Lund et al. (2014) [16]	Denmark	Cohort study	All liveborn singleton infants in Denmark between 1 January 1996 and 31 December 2011	999378	16	19.1
Mohammadizadeh et al. (2010) [17]	Iran	Randomised control trial	Uncomplicated preterm infants (28–34 weeks) born in Shahid-Beheshti and Al-Zahra Hospitals affiliated with the Isfahan University of Medical Sciences	35	0	/

**Fig. 2:**
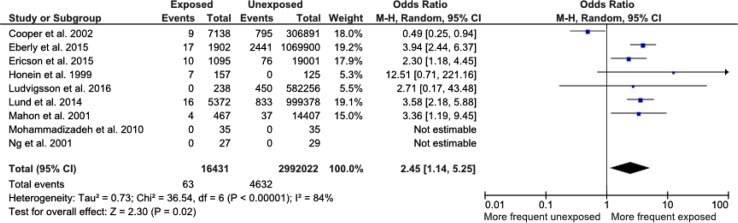
Forest plot comparing the incidence of IHPS between infants with exposure to erythromycin at any time and infants who had never been exposed to erythromycin

**Fig. 3:**
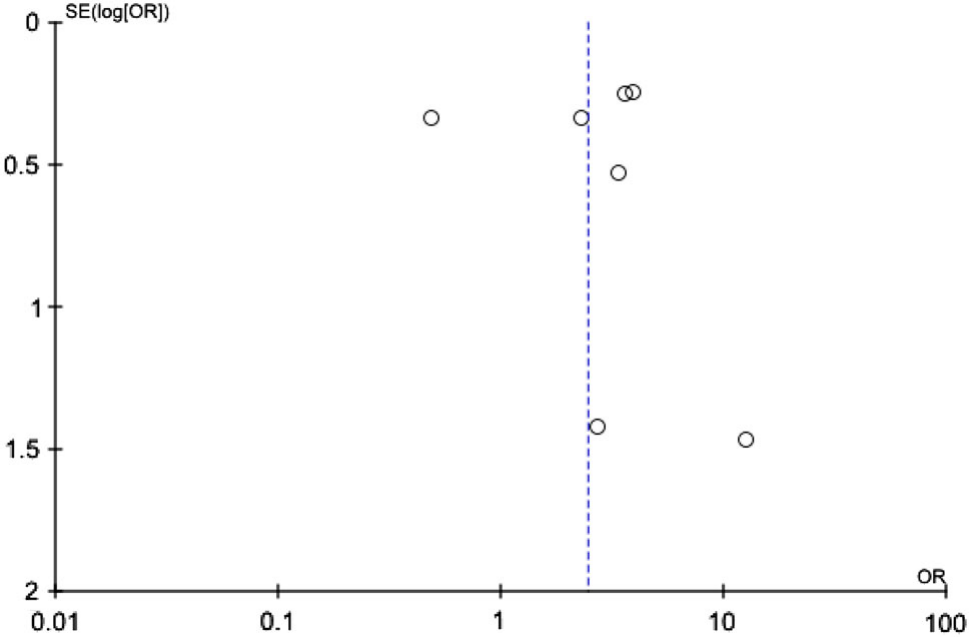
Funnel plot of included studies

Selected cohort studies were published between 1999 and 2016. Cases were defined as infants who developed pyloric stenosis in infancy (age less than 6 months), whilst controls were patients who did not develop pyloric stenosis during the study period. National birth registries, hospital and community health records were the main data sources for both groups. Diagnosis of pyloric stenosis was confirmed from clinical diagnosis recorded in health records. The total number of infants included was 3,008,453, of whom 16,431 had received erythromycin. Sixty-three infants developed IHPS after receiving erythromycin, whereas 4632 infants developed IHPS without having received erythromycin. In the two randomised studies, the total number of infants included was small, and there were no cases of pyloric stenosis in either the exposed or the unexposed groups, so that they could not contribute to the odds ratio. Overall, there was a significant association between erythromycin exposure and subsequent development of pyloric stenosis [OR 2.45 (1.12–5.35), *p* = 0.02, Fig. 2]. However, there was significant heterogeneity between the studies (*I* ^2^ = 84 %, *p* < 0.0001). A funnel plot of published studies demonstrated possible asymmetry indicating potential publication bias, although asymmetry is difficult to determine with only seven studies contributing to the funnel plot (Fig. 3).

**Fig. 4:**
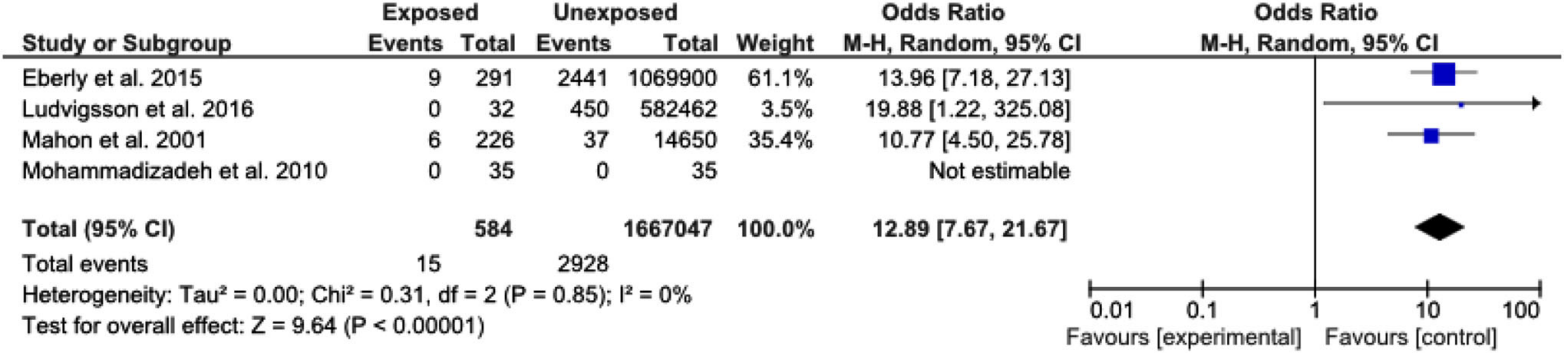
Forest plot comparing the incidence of IHPS between infants with exposure to erythromycin within the first 2 weeks of life and infants who have never been exposed to erythromycin

A further analysis was performed to identify the relationship between exposure to erythromycin in the first 14 days of life and development of IHPS. Only four of the selected nine studies documented whether exposure had occurred within this period. In these studies, the association between erythromycin exposure and subsequent development of pyloric stenosis was even stronger [OR 12.89 (7.67–2167), *p* < 0.00001] (Fig. 4).

## Discussion

This study is the only published meta-analysis which reviews the association between erythromycin use in infants and subsequent development of IHPS and provides a comprehensive estimate of this risk.

The key finding of the meta-analysis is that the OR of developing IHPS after any erythromycin in the post-natal period is two and a half (OR = 2.45) times greater than in those infants not exposed to the drug. Furthermore, subgroup analysis of included studies identified a 12-fold increase in the development of IHPS when erythromycin was administered in the first 14 days of life; a value significantly higher than previously reported.

Literature search did not identify any published meta-analyses and only one systematic review. Maheshwa et al. [7] investigated the relationship between young infants treated with erythromycin and risk of developing hypertrophic pyloric stenosis by analysis of six papers published between 1976 and 2005. Their review concludes that while more evidence is required regarding the relationship between erythromycin use and IHPS, young infants exposed to erythromycin in the first few weeks of life are at a greater risk of IHPS. Their analysis is also in agreement with this study in stating that the risk appears to be highest in the first 2 weeks of life, but stipulates that this occurs in term or near-term infants or when antibiotics are administered for more than 14 days.

It should be noted that two papers included in our analysis, Ng et al. [10] and Mohammadizadeh et al. [17], study populations of preterm infants alone whilst Ericson et al. [15] have analysed only infants within a neonatal intensive care (NICU) environment. Therefore, variability of the calculated OR may occur due to the inclusion of these groups of infants within the analysis. This could also explain the high I^2^ value representing heterogeneity.

In addition, significant geographical bias exists with five of the nine studies selected for analysis focusing on populations from the United States. Such bias may partly result from the literature search criteria which only include studies published in the English language.

A further source of bias occurs due to the greater proportion of cohort studies included for analysis in comparison to other study types. There were no published case–control studies which reviewed this relationship. However, this may result from the ethical feasibility of designing a study which may prevent an infant from receiving erythromycin to treat infection in cases where alternative antibiotics are contraindicated or insensitive.

### Bias

In accounting for the variability of the calculated OR and the significant heterogeneity present between the nine included studies, several factors must be considered. An important factor is that the incidence of IHPS is heterogeneous, varying significantly according to ethnicity, sex and time. There is also a significant genetic component to development of IHPS, so that any conclusive study should also include analysis of confounders, such as gender, ethnicity, and genetic status.

### Risk/benefit

Erythromycin is commonly indicated within the neonatal population for prophylaxis following Chlamydia trachomatis infection [18] in preventing conjunctivitis or pneumonia and in the treatment of pertussis [14]. In addition, erythromycin has also been utilised in the treatment of gastrointestinal dysmotility within this population [10].

Although this study concludes that OR for developing IHPS following erythromycin exposure is high, particularly in the first 14 days of life, physicians must evaluate the risk–benefit ratio in making an informed decision as to whether the potential morbidity or mortality of an infection such as pertussis is outweighed by the risk of developing IHPS. It should also be noted that the absolute risk of developing IHPS following erythromycin exposure is not high [0.4 % (95 % CI 0.3–0.5 %) in those receiving erythromycin at any time, and 2.6 % (95 % CI 1.5–4.2 %) in those receiving erythromycin in the first 14 days]. However, consideration should be made to the fact that despite the indications, macrolides (including erythromycin) remain unlicensed for use by the US Food and Drug Administration for use in infants less than 6 months.

### Limitations

The main limitation of this study is the lack of published studies investigating the relationship between erythromycin use and development of IHPS. Furthermore, differences existed between study designs which may have led to further variability in the calculated ORs. In particular, studies often categorised cases into time periods which often varied between studies resulting in their exclusion despite rigorous methodology. Studies which did not explicitly state that the macrolide administered was erythromycin were also excluded. In addition, all cohort studies included were performed retrospectively, thus having a negative effect on the quality of the data. Our study aimed to exclusively review the effect of neonatal administration of erythromycin on the risk of subsequently developing IHPS. However, the question remains as to whether other methods of exposure (such as maternal administration en-utero or postnatally from absorption via breast milk) may be associated with similar levels of risk. With regard to exposure via breastfeeding, Sorensen [19] concludes that an increased risk of developing IHPS exists following maternal macrolide administration postnatally [OR 2.7 (95 % CI 0.7–11.1)]. However, this is contrasted by two papers by [20] Goldstein et al. and [21] Salman et al. which found no correlation between breast milk exposure and IHPS. The data on exposure via breast milk was too sparse to meta-analyse.

There is also some evidence in the literature that administration of erythromycin to pregnant women may result in the fetus developing IHPS as an infant. Kallen [22] report a risk ratio of 2.51 (95 % CI 0.92–5.46) of infants developing IHPS in cases where their mother had received erythromycin after the first antenatal visit. However, studies by Lin [23] and Louik [24] found no relationship between prenatal exposure to macrolide and pyloric stenosis.

Furthermore, from the papers analysed, there is no report regarding the family history, and therefore it remains unclear if a genetic predisposition is required to increase the risk of acquiring IHPS following administration of erythromycin. With such significant variability in the available literature in reporting the exact nature and magnitude of risk of erythromycin administration (during both fetal and neonatal development) further study is warranted.

## Conclusion

This study provides clinicians with the first comprehensive estimate for the OR of infants developing IHPS when exposed to erythromycin. Physicians should utilise this study as a tool in evaluating the risk–benefit ratio of administering erythromycin for treatment and prophylaxis of infections in neonates versus the risk of developing IHPS. However, in determining whether erythromycin is a suitable treatment for infections within this group, the limitations of this study should be noted. In particular, publications bias and the lack of high-quality, with significant patient numbers should be considered.

## References

[1] Laffolie J, Turial S, Heckmann M, Zimmer KP, Schier F (2012). Decline in infantile hypertrophic pyloric stenosis in Germany in 2000–2008. Pediatrics.

[2] Georgoula C, Gardiner M (2012). Pyloric stenosis a 100 years after Ramstedt. Arch Dis Child.

[3] Krogh C, Fischer TK, Skotte L, Biggar RJ, Øyen N, Skytthe A, Goertz S, Christensen K, Wohlfahrt J, Melbye M (2010). Familial aggregation and heritability of pyloric stenosis. JAMA..

[4] MacMahon B (2006). The continuing enigma of pyloric stenosis of infancy: a review. Epidemiology.

[5] Pedersen RN, Garne E, Loane M, Korsholm L, Husby S, EUROCAT Working Group (2008). Infantile hypertrophic pyloric stenosis: a comparative study of incidence and other epidemiological characteristics in seven European regions. J Matern Fetal Neonatal Med.

[6] Maheshwai N (2007). Are young infants treated with erythromycin at risk for developing hypertrophic pyloric stenosis?. Arch Dis Child.

[7] Mavromati T, Papapanagioutou D, Bursinos B (1995). Gastroduodenal: prokinetic action of erythromycin on the operated stomach. Br J Surg.

[8] Ludvigsson JF, Lundholm C, Örtqvist AK, Almqvist C (2016). No association between macrolide treatment in infancy and later pyloric stenosis in Sweden. Eur J Epidemiol.

[9] Ng PC, So KW, Fung KS, Lee CH, Fok TF, Wong E, Wong W, Cheung KL, Cheng AF (2001). Randomised controlled study or oral erythromycin for treatment of gastrointestinal dysmotility in preterm infants. Arch Dis Child Health Fetal Neonatal Ed.

[10] Cooper WO, Griffin MR, Arbogast P, Hickson GB, Gautam S, Ray WA (2002). Very early exposure to erythromycin and infantile hypertrophic pyloric stenosis. Arch Pediatr Adolesc Med.

[11] Eberly MD, Eide MB, Thompson JL, Nylund CM (2015). Azithromycin in early infancy and pyloric stenosis. Paediatrics.

[12] Mahon BE, Rosenman MB, Kleiman MB (2001). Maternal and infant use of erythromycin and other macrolide antibiotics as risk factors for infantile hypertrophic pyloric stenosis. J Pediatr.

[13] Honein MA, Paulozzi LJ, Himelright IM, Lee B, Cragan JD, Patterson L, Correa A, Hall S, Erickson JD (1999). Infantile hypertrophic pyloric stenosis after pertussis prophylaxis with erythromycin: a case review and cohort study. Lancet.

[14] Ericson JE, Arnold C, Cheeseman J, Cho J, Kaneko S, Wilson E, Clark RH, Benjamin DK, Chu V, Smith PB, Hornik CP, Best Pharmaceuticals for Children Act-Pediatric Trials Network Administrative Core Committee (2015). Use and safety of erythromycin and metoclopramide in hospitalized infants. J Pediatr Gastroenterol Nutr..

[15] Lund M, Pasternak B, Davidsen RB, Feenstra B, Krogh C, Diaz LJ, Wohlfahrt J, Melbye M (2014). Use of macrolides in mother and child and risk of infantile hypertrophic pyloric stenosis: nationwide cohort study. BMJ.

[16] Mohammadizadeh M, Ghazinour M, Iranpour R (2010). Efficacy of prophylactic oral erythromycin to improve enteral feeding tolerance in preterm infants: a randomised controlled study. Singapore Med J..

[17] Rosenman MB, Mahon BE, Downs SM, Kleiman MB (2003). Oral erythromycin prophylaxis vs watchful waiting in caring for newborns exposed to *Chlamydia trachomatis*. Arch Pediatr Adolesc Med.

[18] Sørensen HT, Skriver MV, Pedersen L, Larsen H, Ebbesen F, Schønheyder HC (2003). Risk of infantile hypertrophic pyloric stenosis after maternal postnatal use of macrolides. Scand J Infect Dis.

[19] Goldstein LH, Berlin M, Tsur L, Bortnik O, Binyamini L, Berkovitch M (2009). The safety of macrolides during lactation. Breastfeed Med.

[20] Salman S, Rogerson SJ, Kose K, Griffin S, Gomorai S, Baiwog F, Winmai J, Kandai J, Karunajeewa HA, O’Halloran SJ, Siba P, Ilett KF, Mueller I, Davis TM (2010). Pharmacokinetic properties of azithromycin in pregnancy. Antimicrob Agents Chemother.

[21] Källén BA, Otterblad Olausson P (2005). Danielsson BR (2005) Is erythromycin therapy teratogenic in humans?. Reprod Toxicol.

[22] Lin KJ, Mitchell AA, Yau WP, Louik C, Hernández-Díaz S (2013). Safety of macrolides during pregnancy. Am J Obstet Gynecol.

[23] Louik C, Werler MM, Mitchell AA (2002). Erythromycin use during pregnancy in relation to pyloric stenosis. Am J Obstet Gynecol.

